# Emotional prosody recognition enhances and progressively complexifies from childhood to adolescence

**DOI:** 10.1038/s41598-022-21554-0

**Published:** 2022-10-13

**Authors:** M. Filippa, D. Lima, A. Grandjean, C. Labbé, S. Y. Coll, E. Gentaz, D. M. Grandjean

**Affiliations:** 1 Faculty of Psychology and Educational Sciences, Swiss Center of Affective Sciences, University of Geneva, Geneva, Switzerland; 2 Neuroscience of Emotions and Affective Dynamics Laboratory, Unimail, University of Geneva, Geneva, Switzerland; 3 Educational Medical Center of Boissonas, Office Médico-Pédagogique, Geneva, Switzerland; 4 Neurorehabilitation Division, Beau-Séjour Hospital, 26 Av. de Beau-Séjour, 1211 Geneva 14, Switzerland

**Keywords:** Psychology, Health care, Risk factors

## Abstract

Emotional prosody results from the dynamic variation of language’s acoustic non-verbal aspects that allow people to convey and recognize emotions. The goal of this paper is to understand how this recognition develops from childhood to adolescence. We also aim to investigate how the ability to perceive multiple emotions in the voice matures over time. We tested 133 children and adolescents, aged between 6 and 17 years old, exposed to 4 kinds of linguistically meaningless emotional (anger, fear, happiness, and sadness) and neutral stimuli. Participants were asked to judge the type and intensity of perceived emotion on continuous scales, without a forced choice task. As predicted, a general linear mixed model analysis revealed a significant interaction effect between age and emotion. The ability to recognize emotions significantly increased with age for both emotional and neutral vocalizations. Girls recognized anger better than boys, who instead confused fear with neutral prosody more than girls. Across all ages, only marginally significant differences were found between anger, happiness, and neutral compared to sadness, which was more difficult to recognize. Finally, as age increased, participants were significantly more likely to attribute multiple emotions to emotional prosody, showing that the representation of emotional content becomes increasingly complex. The ability to identify basic emotions in prosody from linguistically meaningless stimuli develops from childhood to adolescence. Interestingly, this maturation was not only evidenced in the accuracy of emotion detection, but also in a complexification of emotion attribution in prosody.

## Introduction

Emotional prosody can be defined as the ensemble of segmental and supra-segmental variations (referring to melodic aspects) of our speech production during an emotional experience, and it is conceived as an interface between language and affect^1^. Emotional prosody categories have been described as correlating with a range of acoustic features which are essentially musical: rhythm, pitch, tone, amplitude, accent, pause, duration^2^, and their unfolding. Each vocal emotion has its own acoustic profile, and the ability to decode emotions during social exchanges is not only crucial for developing social abilities, but is necessary for establishing fundamental affiliations in infancy and intimate relationships during development and in life^3,4^. The vocal communication of emotions is thought to follow a model of dyadic processes, which are determinant for accurate encoding (or production) and decoding (or recognition) of vocal affects during social exchanges^5–7^. In these processes, prosodic features of vocal production play a fundamental role in decoding partners’ emotions^8^ and is a key index for assessing children, adolescents, and adults’ affective abilities.

### The development of emotion recognition

Basic recognition and knowledge of emotions develop early in life and grows throughout childhood and adolescence, improving our understanding, ability to manage, and adaptively utilize emotions in crucial periods of development^9,10^.

Visual and auditory sensory abilities play a crucial role in the early development of emotion recognition from faces and voices, respectively. Visual and auditory emotional information are related and both support early multimodal recognition of emotions as is the case in adults^11^. In the newborn period, facial recognition of an intimate partner is likely rooted in a prior experience with the mother’s voice, the latter being a highly salient and detectable signal even during pregnancy^12,13^. During infant and child development, senses operate together to convey and to process emotional information, and the role of redundancy in cross-modal expression and perception of emotions is crucial for their emotional development^14–16^.

Emotion recognition, especially in childhood and adolescence, is deeply linked with emotion regulation, which leads to better school performance and to improved relationships with teachers in school^17^. Higher levels of emotion knowledge lead to better social skills in childhood and adolescence^18^ and, later in life, is a strong predictor of effective social behavior as well as early school and later academic success^19–21^.

While facial recognition of non-verbal cues has been broadly investigated from a developmental perspective^22–24^, the origins and development of vocal emotion recognition from childhood to adolescence has been less investigated^25^.

### The development of emotion recognition in vocalizations

Though less investigated than facial emotion recognition, children’s and adolescents’ ability to recognize emotions from voices has been the object of several studies.

In their systematic review^26^, Morningstar and colleagues report that the ability to detect emotions in linguistic stimuli begins very early^27^ and it improves with the age over childhood^2,9,28–31^.

From a cross-cultural perspective, Chronaki et al.^32^ demonstrated not only the universality of vocal emotion recognition in children, but also that native English-speaking children showed higher accuracy in recognizing vocal emotions in their native language, with a larger improvement during adolescence. The vocal stimuli were linguistic utterances in their native language (English) and foreign languages (Spanish, Chinese, and Arabic).

It is obvious that familiarity with the linguistic stimulus, not only involves semantic meaning processing, but constitutes an important factor contributing to the vocal processing of emotions. For this reason, some interesting studies have also been performed using non-linguistic vocal stimuli.

Matsumoto and Kishimoto^33^ demonstrated that Japanese children begin to correctly recognize all basic emotions from nonverbal vocal cues from 7 to 9 years of age. The stimuli were the first 15 syllables of the Japanese syllabus performed with emotional content by professional actors.

Chronaki et al.^9^ asked 4–11‐year‐old children to recognize emotions from non‐word vocal stimuli (‘ah’ interjection) and reported an improvement in emotion recognition with age, with a continuing development in late childhood.

Sauter and colleagues^28^, used vocal non-speech sounds such as laughs, sighs, and grunts, asking children to associate vocalizations with facial expressions in pictures in a four-way forced choice task. Children as young as 5 years old could reliably infer emotions from non-verbal vocal cues. However, this recognition did not improve significantly with age, probably due to an early ability to associate laughs, sighs, and grunts to the correct facial expression. This was not the case for linguistic stimuli (emotionally inflected speech), which were better recognized as age increased.

Allgood and Heaton^29^, using the same stimuli as in Sauter^34^ laughs, sighs, and grunts—showed an age-related increase in the ability to recognize emotions in 5–10-year-old children.

Finally, Grosbras et al.^35^ used vocal bursts expressing four basic emotions and asked children and adolescents to detect the correct emotion in a forced choice task. The ability to recognize emotions in nonlinguistic utterances increased with age and was driven by anger and fear recognition. Between 14 and 15 years of age, adolescents reached adult performances in emotion recognition, and across ages, girls obtained better scores than boys for several emotions.

Interjections, short vocal non-speech sounds and vocal bursts have thus been chosen as non-linguistic stimuli to investigate the development of emotion recognition in voices.

The novelty of the present study lies primarily in the choice of meaningless speech stimuli. Pseudo-sentences made of pseudowords that respect linguistic rules such as syllabic and word organization^36–41^, which do not convey a semantic content but keep prosodic information intact. Thus, we were both consistent with linguistic stimuli studies by deciding to concentrate on emotional prosody and with non-linguistic studies by avoiding the effect of linguistic semantic information.

### The concept of complexification in emotion recognition

The maturation of the ability to recognize emotions from behavioral cues does not only manifest in an increased ability to recognize and experience emotions, but also in an improved capacity to perceive multiple emotions in a stimulus.

In real life, people express emotions using acoustic characteristics pertaining to two or more basic emotions and the ability to detect emotions becomes more complex throughout development. In fact, while children of 5–6 years of age tend to perceive and experience single, often polarized emotions (e.g., good and bad)^42^, as they grow up there is a tendency for emotional experiences to become more complex, mixed, or even contradictory^43^.

### Aims and hypotheses

The primary objective of the present study was to investigate if children’s ability to recognize emotions in prosody from meaningless vocal stimuli improved with age. For this, we used long meaningless emotionally expressive vocal stimuli, using multiple choice and continuous scales (see methods). Secondly, we investigated how children and adolescents attributed multiple emotions to the vocal stimuli, in presence of a correct response. For the latter, we tested whether the representation of emotions perceived in affective vocal prosody became progressively complex in children and young adults through the use of continuous scales. As the ability to feel multiple emotions increases with age, we posited that similar trajectories would also manifest in the recognition of multiple emotions in vocal prosody.

## Methods

### Participants and procedure

133 participants (58 males) between 6 and 17 years old (*M* = 11.32; *SD* = 5.6) were recruited from La Salle primary school in Thonon-les-Bains, France.

All experimental protocols were approved by the University of Geneva Ethics Committee, and all methods were carried out in accordance with relevant guidelines and regulations. Finally, informed consent was obtained from all subjects’ legal guardians.

Participants were tested on individual laptops, the stimuli were presented through headphones, and the responses were made through ratings on continuous scales with a cursor. The testing phase was preceded by an initial training where participants listened to bilaterally presented stimuli through a homemade Authorware program. Answers were considered correct when the target emotion was rated higher than other emotions on visual analog scales^44^. In addition, participants had the option of responding “I don't know” and could listen to the emotional stimuli up to three times maximum.

### Stimuli

Participants were asked to judge four basic vocal emotions (joy, fear, anger, and sadness) and neutral stimuli expressed by adult voices. Judgments were made on six different visual analog continuous scales: joy, fear, sadness, anger, neutral, and surprise.

Stimuli composed of pseudowords constituting pseudosentences from the GEMEP (Geneva Multimodal Emotion Portrayals) corpus^37^ and the Munich database^45^ were used.

The 30 vocal stimuli (mean duration 2044 ms, from 1205 to 5236 ms) were pseudo-randomly (avoiding more than three consecutive stimuli of the same category) assigned to two different lists. The pseudo-randomization process was carried out with respect to the duration, the mean acoustic energy, and the standard deviation of the mean energy of each sound sample.

The mean duration of the stimuli was 2044 ms (Range: 1205–5236 ms). No significant differences in duration were found between prosodic categories (*F*(4, 156) = 1.43, *p* > .10); and no significant difference was found in mean acoustic energy of the samples, *F*(4, 156) = 1.86, *p* > .10. Likewise, there was no significant difference between categories for the standard deviation of the mean energy of the sound stimuli, *F*(4, 156) = 1.9, *p* > .10.

Using meaningless utterances allowed us to avoid the potential impact of meaningful lexical-semantic information upon perceiving vocally expressed emotions (see Appendix 1 for some examples of the adopted stimuli). We used the pseudoutterances of these corpora, which were based on European languages (for syllabic and word organization) to avoid a confounding semantic effect.

### Analyses and statistics

We performed General or Generalized linear mixed models using R (version 4.0.0) in RStudio (version 1.2.5042)^46^. Models included three fixed factors: Target emotions (five modalities: anger, happy, neutral, fear, and sadness), Scale (six modalities: anger, happy, neutral, fear, sadness, and surprise), Age (as a continuous variable), and two random factors (user ID and corpus version: GEMEP and Munich corpora). We systematically tested the more complex model (e.g. for the full model: main effects and the interactions with Age, Emotion presented, and Scale) with the relevant simpler model (e.g. main effects plus two-way interactions), then the chi-square test either did or did not reveal a significant increase in explained variance for the more complex model (e.g. with the adding of the three-way interaction). For the first analysis, in order to identify the correct responses, we discretized the response as correct (1) or incorrect (0) according to the continuous scale scores for each trial. The response was discretized as ‘correct’ if the participant’s score on the target scale was the highest (e.g. highest value on the fear scale in response to a fearful vocalization). In no case did we have the same rating in two different scales. Therefore, we did not have to make choices to identify the correctness of the response. Then we used Generalized linear mixed models specifying a binomial family. To test the significant increase or decrease of emotion recognition with Age, we tested to what extent the slope of the percentage of correct responses with Age was different from 0. For the complexification hypothesis we used the sum of the values judged on non-target scales using only the correct trials (those with the highest values on the target scale). Then we predicted an increase of the sum on the non-target scales, as an indicator of more complex emotion attribution, with Age. For contrast analysis, we used the emmeans R package. We corrected the *p*-values using Bonferroni multiple correction when the tests were not independent (e.g. for non-target emotion, corrected *p* value = 0.5/6 = .0083). The datasets generated during the current study are available from the corresponding author upon request.

### Ethical approval

University of Geneva Ethics Committee.

## Results

### Age and sex effect

**Figure 1:**
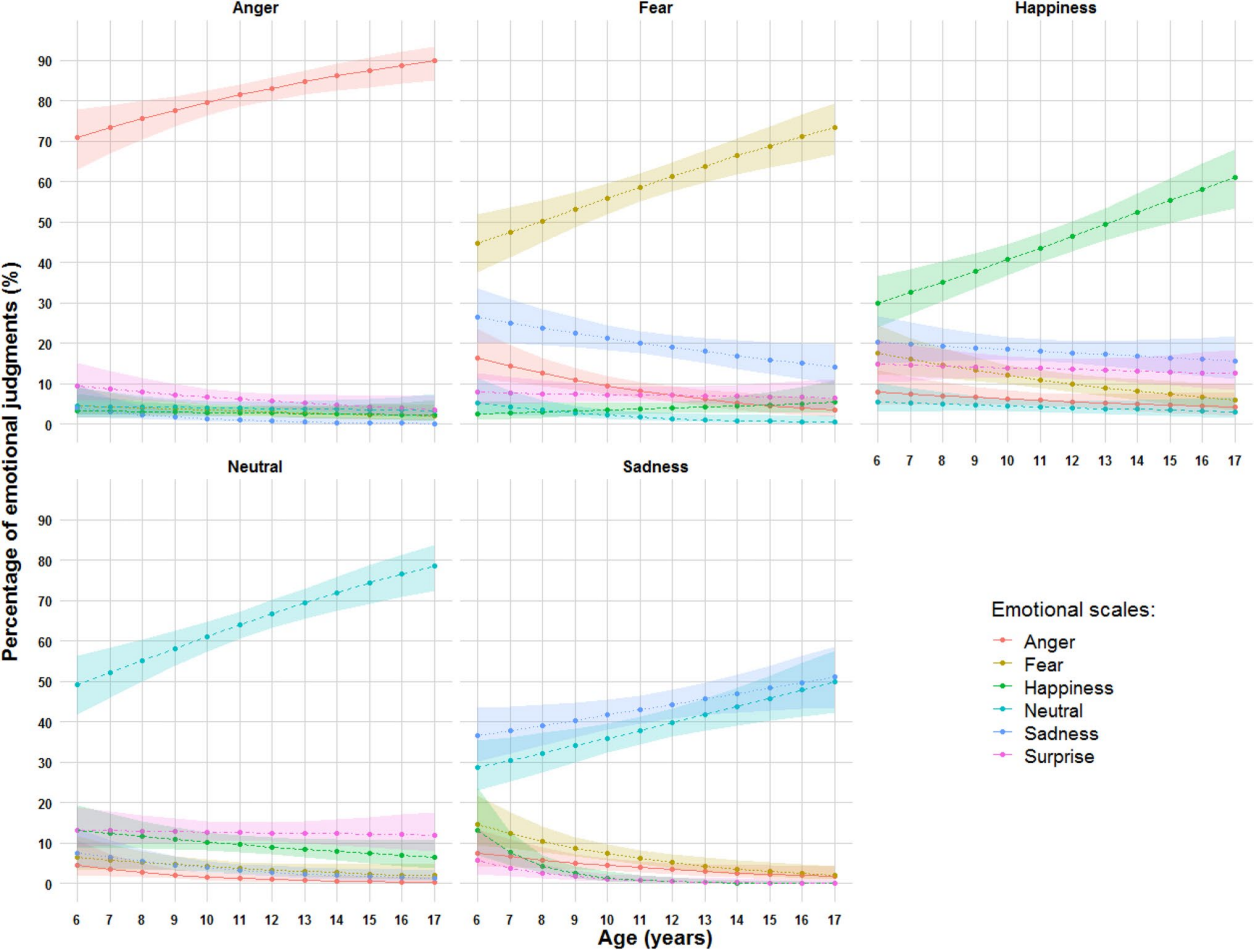
Percentages of emotion judgments as a function of age. Shaded areas represent 95% confidence intervals. The percentage of correct responses homogeneously increased with age.

The general effect of Age was not significant (χ^2^ (1) = 1.01, *p* = .310), but, as predicted, the interaction between Age, Emotion, and Scale revealed that children’s ability to correctly recognize the target emotion increased with Age (χ^2^ (1) = 224.56, *p* < .001, see Fig. 1).

In particular, the percentage of correct responses significantly increased with Age for Anger (χ^2^ (1) = 13.22, *p* < .001), Happiness (χ^2^ (1) = 22.75, *p* < .001), Neutral (χ^2^ (1) = 22.30, *p* < .001), and Fear (χ^2^ (1) = 19.96, *p* < .001). The test of the slope against zero for Sadness (χ^2^ (1) = 5.05, *p* = .025) did not reach the corrected *p* value (*p* = .008).

The percentage of responses for the presented emotions is reported in Appendix 1, Table A1.

All other tested comparisons are reported in Appendix 1, Table A2.

As Age increases, Fear was less confused with Happiness, Neutral, and Sadness (i.e., for Happiness, χ^2^ (1) = 8.21, *p* = .004), and Neutral was less confused with Sadness (χ^2^ (1) = 8.25, *p* = .004). The confounder, Surprise, tended to remain stable across Ages and across target Emotions.

The general effect of Sex was marginally significant across ages and emotions (*p* = .069). However, in the specific Age and Emotion interaction, there was a significantly better performance of girls compared to boys in recognizing Anger (χ^2^ (1) = 3.88, *p* = .049). Moreover, boys rated Fear as Neutral, significantly more than girls did (χ^2^ (1) = 4.75, *p* = .029). For a graphical representation of the evolution of correct responses, see Appendix Figure A1.

### Emotion effect

The percentage of correct responses homogeneously increased with Age across Emotions (*p* < 2.2 × 10^−16^, for details see Appendix, Table A1). This increase with age, calculated with the slope contrasts, was not significantly different across emotions, except for the contrasts between Anger, Happiness, and Neutral versus Sadness slopes that were marginally significant (Anger/Sadness: χ^2^ (1) = 2.88, *p* = .089; Happiness/Sadness: χ^2^ (1) = 3.32, *p* = .068; Neutral/Sadness: χ^2^ (1) = 3.64, *p* = .056). When comparing the means of the corrected responses without the Age groups, Anger was the emotion recognized with the highest accuracy in prosody, followed by Neutral and Fear. Happiness and Sadness were around the same level, being less well recognized than the others (see Appendix, Figure A1 and Appendix, Table A1 for the mean values, as well as Appendix, Table A2 for systematic contrasts).

### Multiple emotion recognition

**Figure 2:**
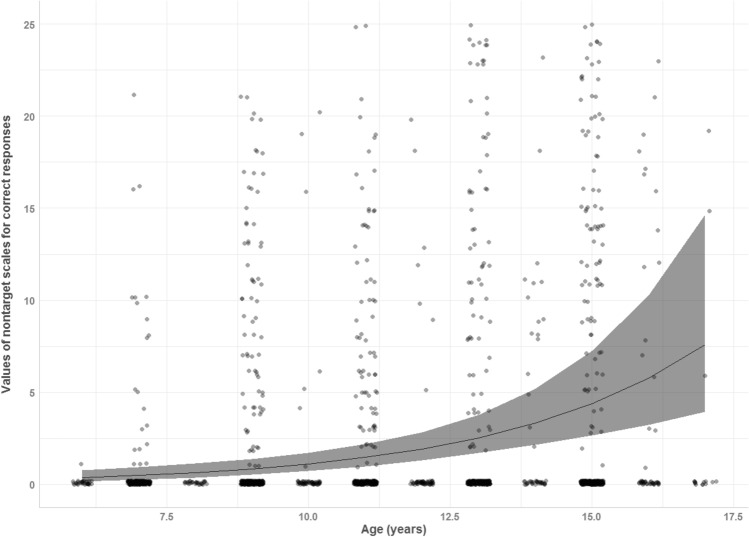
Proportion of correct responses as a function of age and sex.

Even in the presence of a correct detection of the target emotion, participants added other emotions as being present in the vocal prosody extracts. These additional emotion attributions significantly increased with age (χ^2^ (1) = 26.18, *p* < .001). Results reported in Fig. 2 show that multiple emotion recognition increased non-linearly between age groups. For example, 6–7 year-old children did not differ from the 8–9 year-olds (corrected *p* value is .013; *t*(1) = − 2.38, *p* = .018), and 8–9 year-old children did not differ from the 10–11 year-olds (*t*(1) = 0.6, *p* = .550). However, 10–11 year-olds and 12–13 year-olds showed higher multiple emotion recognition levels than the 6–7 year-olds (*t*(1) = 2.92, *p* = .004; and *t*(1) = 3.35, *p* = .001 respectively), and the same thing happened with 14–17 year-old children compared to the 8–9 year-olds (*t*(1) = 2.73, *p* = .006).

## Discussion

The main aim of the present study was to understand how the recognition of emotional prosody develops from childhood to adolescence. Emotional prosody recognition was tested in children using linguistically meaningless stimuli (pseudoutterances), allowing us to keep the prosodic aspect of the sentence intact, but without semantic information. We tested recognition by having participants judge the intensity of all emotions on separate continuous scales (no forced choice on one single emotion). This last original methodological choice allowed us to measure the presence or absence of multiple perceived emotions in each single stimulus.

First, we demonstrated that participants’ ability to correctly recognize the target emotion in prosody improved with age, from childhood to adolescence. This was true for all tested emotions except for the recognition of sadness, which was stable across ages.

Our findings on sadness perception are consistent with previous results reporting that young children have difficulty recognizing sadness in voices and that sadness recognition from facial expressions is delayed across development^9,33^, except for one study where sadness was expressed by cry vocalizations^35^. In his review on the development of face and voice processing during infancy and childhood, Grossman examines the event-related potential correlates of emotion processing in the voice during infancy^27^. When discussing his data in light of adult studies, he concludes that infants and children allocate more attentional resources to angry than to happy or neutral voices. This last observation may partially explain why in the present study it is more difficult for children and young adults to correctly identify sadness than anger.

However, apart from sadness, all other basic emotions were identified with equal accuracy. This aspect is not in line with previous research reporting, for example, that across age groups happiness is the easiest emotion to recognize^35^. This may be due to the fact that in our study the stimuli were based on a linguistic structure and that they were longer and more complex than vocal bursts^35^ or non-speech sounds, such as laughs, sighs, and grunts^28^.

Secondly, the present study demonstrates that girls tend to recognize anger better than boys, and boys confuse fear and neutral stimuli significantly more than girls do.

These results are in line with the literature confirming that girls, across ages, are slightly favored in encoding the nonverbal elements of emotion expression in voices and faces^47–49^. There is also a potential increase in the size of this advantage from childhood to early adulthood^50^. In our study we found a specific ability of girls to recognize negative emotions, such as anger. This is in line with Grosbras et al.^35^ who also found sex differences between adolescent boys and girls in the identification of basic emotions in vocal bursts, in particular for fear. Also, in the present study, fear is less confused by girls and this result is in line with studies on the development of facial emotion recognition^51^. Taken together, the present results are consistent with most of the literature in confirming that girls show better and more accurate detection of negative emotions, such as anger and fear. This could be partly explained by the theory that during evolution women had to develop stronger self-protective reactions than men to cope with aggressive behaviors, such as anger and fear-related behaviors^52,53^. Whether the emotion recognition of anger and fear also shows specific different neural correlates during emotion processing is still unknown, and could be the object of future studies.

Finally, the present study demonstrates that participants are significantly more likely to attribute multiple emotions to emotional prosody with age, showing that young adults’ emotion representation of perceived emotional prosody becomes progressively complex.

One of the indexes for evaluating emotion maturation is the increased ability to experience and to recognize multiple emotions in others. During childhood there is an evident tendency to feel and to attribute a single emotion. This tendency becomes gradually complex during development^54^. Children between 3 and 6 years of age demonstrate an initial capacity to both experience and understand mixed emotions^55^. This ability gradually develops, together with the ability to experience complex and possibly contradictory mixed emotions, as for example in the context of sarcasm or irony in complex social interactions. It is also possible that the differences between younger and older children in recognizing multiple emotions are mediated by developmental differences in empathy, the ability to experience others’ emotions. To our knowledge, this complexification perspective, positing that there is a continuity in the emotional development of children, has never been tested for prosody. In the present study, thanks to non-forced-choice emotion ratings, we demonstrated that this complexification of the emotional construct is also evidenced in vocal emotion recognition and that it gradually matures during adolescence. Specifically, our results suggest it takes at least 2–3 years for emotion recognition from prosody to become more complex and to show a significant increase in multiple emotion detection values. Further in line with the view of continuity in emotion development from childhood, adolescents gradually improve their ability to decode multiple emotions in prosody at least up to 12–14 years old. Further studies are needed to determine whether the developmental increase in the understanding and experiencing of multiple and contradictory emotions also develop during the lifespan.

One limitation of the present study is that the vocal stimuli were created by adult actors and were not pre-rated in a younger population of adolescents and children. As developmental changes in vocal emotion recognition may depend on the age of the speaker, adolescents being less accurate when identifying emotional prosody presented by other youth^26^, future research should test children’s ability to detect emotion in voices when presented by adults or by children.

## Conclusions

To conclude, our study demonstrates that the ability to identify basic emotions from emotional prosody, using linguistically meaningless stimuli, thus not related to their semantic content, develops from childhood to adolescence. Interestingly, this maturation was not only evidenced in the accuracy of emotion detection, but also in emotion attribution to prosody becoming more complex. Understanding emotions from emotional prosody is crucial during interactions and deepening our understanding of others’ emotions allows for a more flexible adaptation to others’ intentions and to plural social demands.

However, few studies are conducted on the neural mechanisms that might contribute to this maturation process. Potentially, the brain areas involved in adult vocal perception may show age-related changes, especially from childhood to adolescence, underpinning their capacity to recognize emotions from prosody in linguistically meaningless stimuli.

Future research should investigate the neural correlates of age-related improvement in emotional prosody recognition and the neural basis of the emergence of complexification in emotion recognition during adolescence. Prospectively, a detailed acoustic analysis of the vocal stimuli could allow us to understand the acoustic factors leading to misunderstandings in the emotional prosody or to the complexification of emotion recognition in voices.

## Supplementary Information

Supplementary Information 1.

Supplementary Information 2.

Supplementary Information 3.

Supplementary Information 4.
