# Supplementary material for: Emotional prosody recognition enhances and progressively complexifies from childhood to adolescence
Source: Sci Rep. 2022 Oct 13;12:17144. doi: 10.1038/s41598-022-21554-0 (PMC9561714; doi:10.1038/s41598-022-21554-0)
Supplement: Supplementary file 1 — Supplementary Information 1. [file 41598_2022_21554_MOESM1_ESM.docx]

**Appendix 1**

Table A1. Percentage of responses for the five presented emotions across the different age groups. In bold, the percent of correct ratings (Anger vocal prosody rated as Anger, etc.).

|  | **6-7 years (n=23)** | | | | | **8-9 years (n=25)** | | | | | **10-11 years (n=24)** | | | | | **12-13 years (n=26)** | | | | | **14-17 years (n=35)** | | | | |
| --- | --- | --- | --- | --- | --- | --- | --- | --- | --- | --- | --- | --- | --- | --- | --- | --- | --- | --- | --- | --- | --- | --- | --- | --- | --- |
|  | *Mean_correct response_*= 44.32 | | | | | *Mean_correct response_ =* 56.12 | | | | | *Mean_correct response_ =*56.08 | | | | | *Mean_correct response_ =*63.42 | | | | | *Mean_correct response_ =*64,44 | | | | |
| **Emotions** | **Presented** | | | | | **Presented** | | | | | **Presented** | | | | | **Presented** | | | | | **Presented** | | | | |
| **Responses** | **A** | **H** | **N** | **F** | **S** | **A** | **H** | **N** | **F** | **S** | **A** | **H** | **N** | **F** | **S** | **A** | **H** | **N** | **F** | **S** | **A** | **H** | **N** | **F** | **S** |
| **A**nger | **69.3** | 8.8 | 1.5 | 15.3 | 5.2 | **83.3** | 7.2 | 4.6 | 11.4 | 6.5 | **72.1** | 2.7 | 1.4 | 4.1 | 2.7 | **86.6** | 5.0 | 0 | 7.6 | 4.4 | **86.4** | 5.7 | 0.5 | 5.1 | 1.9 |
| **H**appiness | 3.6 | **36.8** | 19.9 | 2.2 | 10.4 | 2.0 | **34.2** | 5.3 | 2.5 | 0.7 | 4.1 | **38.4** | 7.5 | 5.5 | 0 | 0.6 | **45.3** | 4.5 | 3.2 | 0.6 | 3.3 | **58.8** | 10.8 | 4.6 | 0.5 |
| **N**eutral | 3.6 | 5.9 | **41.2** | 6.6 | 23.7 | 2.0 | 4.6 | **66.9** | 0.6 | 40.5 | 6.8 | 2.1 | **63.3** | 0.7 | 37.7 | 6.4 | 6.3 | **75.0** | 1.3 | 40.5 | 1.4 | 2.8 | **67.6** | 1.4 | 44.1 |
| **F**ear | 5.8 | 13.2 | 8.1 | **36.5** | 12.6 | 2.0 | 16.4 | 2.6 | **56.3** | 8.5 | 2.7 | 9.6 | 2.7 | **63.4** | 5.5 | 3.2 | 11.9 | 2.6 | **64.6** | 3.8 | 2.3 | 5.7 | 3.3 | **63.0** | 4.3 |
| **S**adness | 3.6 | 16.9 | 8.1 | 26.3 | **37.8** | 0.7 | 19.7 | 3.3 | 21.5 | **39.9** | 2.0 | 25.3 | 2.0 | 20.7 | **43.2** | 0 | 12.6 | 3.2 | 13.9 | **45.6** | 0.5 | 15.6 | 1.9 | 17.1 | **46.4** |
| **Surprise** | 8.8 | 12.5 | 14.0 | 9.5 | 4.4 | 8.0 | 13.2 | 11.9 | 7.0 | 1.3 | 7.5 | 16.4 | 13.6 | 4.1 | 0 | 1.9 | 17.0 | 9.0 | 7.0 | 1.3 | 5.2 | 10.0 | 13.6 | 7.4 | 0 |
| **I don’t know** | 5.1 | 5.9 | 7.4 | 3.6 | 5.9 | 2.0 | 4.6 | 5.3 | 0.6 | 2.6 | 4.8 | 5.5 | 9.5 | 1.4 | 11.0 | 1.3 | 1.9 | 5.8 | 2.5 | 3.8 | 0.9 | 1.4 | 2.3 | 1.4 | 2.8 |

Table A2. Values, Chi-squared and p values for the contrasts of the five presented emotions with the six recognized emotions. In bold, the significant corrected p values (<.0083).

| **Emotions** | **Anger** | | | **Happiness** | | | **Neutral** | | | **Fear** | | | **Sadness** | | | |
| --- | --- | --- | --- | --- | --- | --- | --- | --- | --- | --- | --- | --- | --- | --- | --- | --- |
| **Responses** | Value | χ^2^(1) | p | Value | χ^2^(1) | p | Value | χ^2^(1) | p | Value | χ^2^(1) | p | Value | χ^2^ | p |  |
| **Anger** | .23 | 13.22 | **3x10^-4^** | -.11 | 1.14 | .29 | -.55 | 5.88 | .015 | -.31 | 11.55 | **6x10^-4^** | -.31 | 5.34 | 0.02 |  |
| **Happiness** | -.04 | .08 | .77 | .24 | 22.75 | **2x10^-6^** | -.15 | 3.16 | .076 | .14 | 1.18 | 0.28 | -1.59 | 10.93 | **9x10^-4^** |  |
| **Neutral** | -.02 | .03 | .86 | -.10 | .73 | .39 | .24 | 22.30 | **2x10^-6^** | -.69 | 7.13 | **0.007** | .16 | 10.74 | **0.001** |  |
| **Fear** | -.18 | 1.61 | .20 | -.22 | 8.21 | **.004** | -.23 | 3.13 | .077 | .22 | 19.96 | **8x10^-6^** | -.39 | 14.09 | **2x10^-4^** |  |
| **Surprise** | -.21 | 4.0 | .04 | -.03 | .26 | .61 | -.23 | .10 | .755 | -.01 | .003 | 0.96 | -.87 | 7.99 | **0.004** |  |
| **Sadness** | -.72 | 5.39 | .02 | .06 | .81 | .37 | -.51 | 8.25 | **.004** | -.14 | 5.59 | 0.01 | .11 | 5.05 | 0.02 |  |

Figure A1. Proportion of correct responses as a function of age and sex.


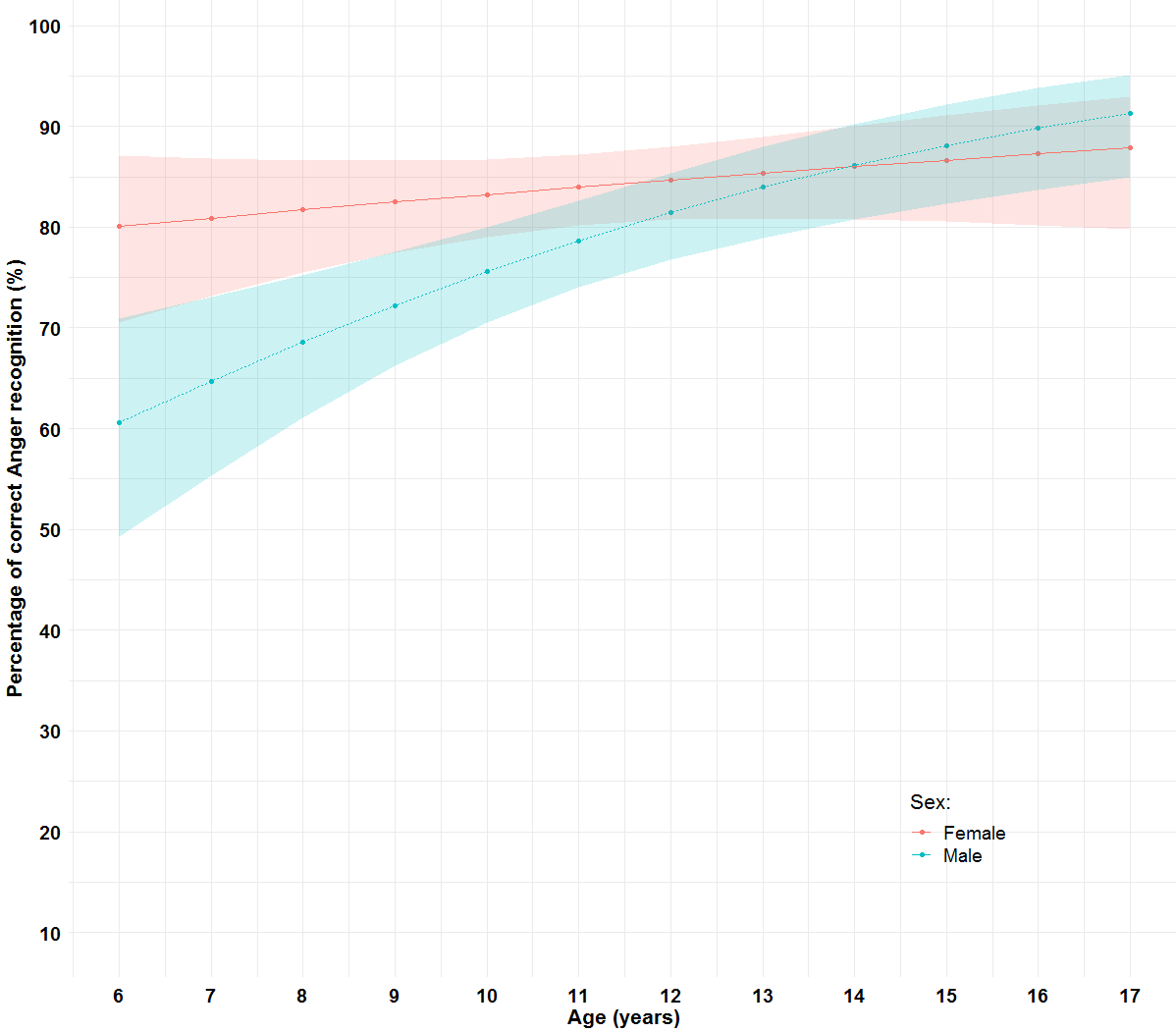

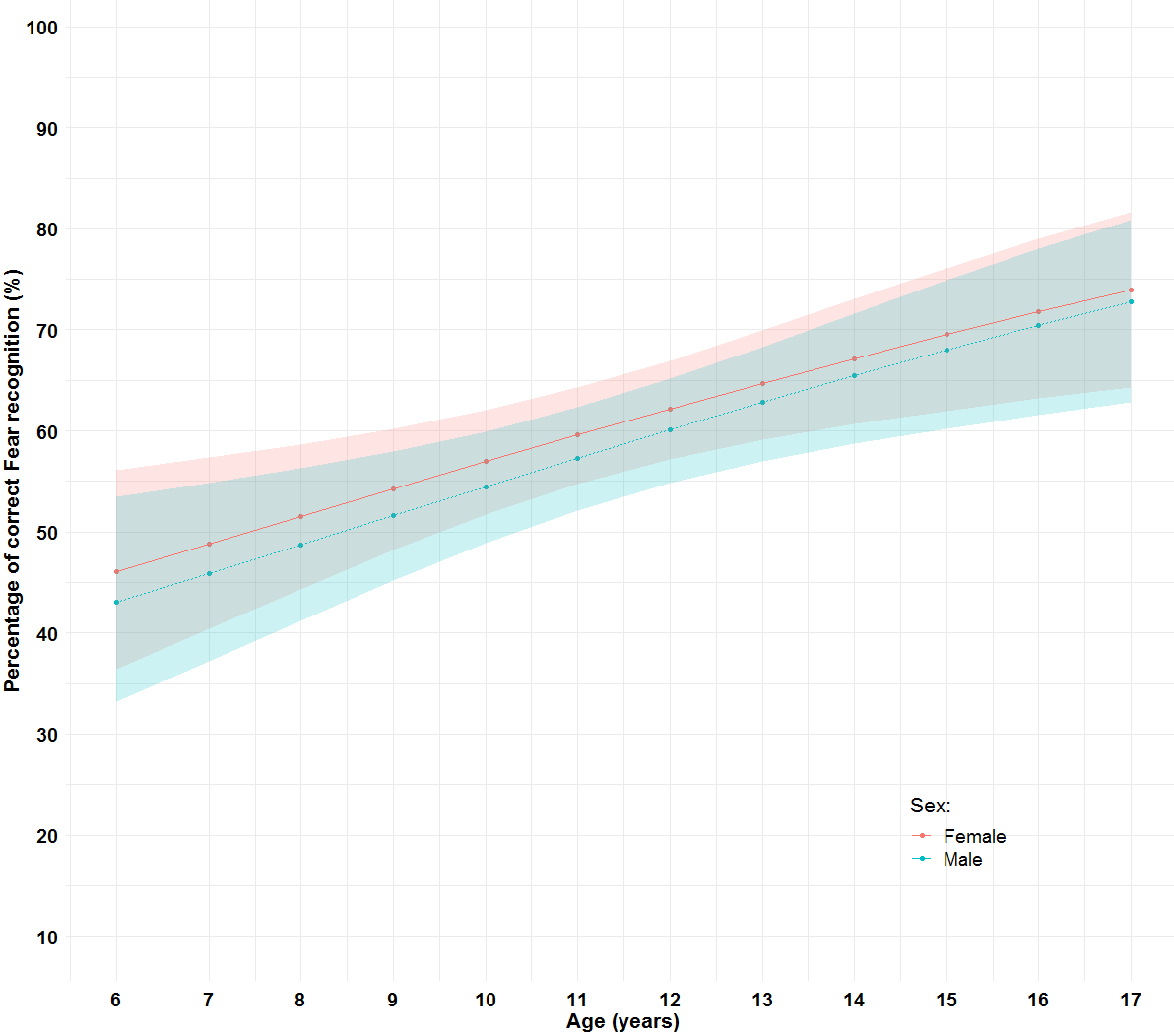

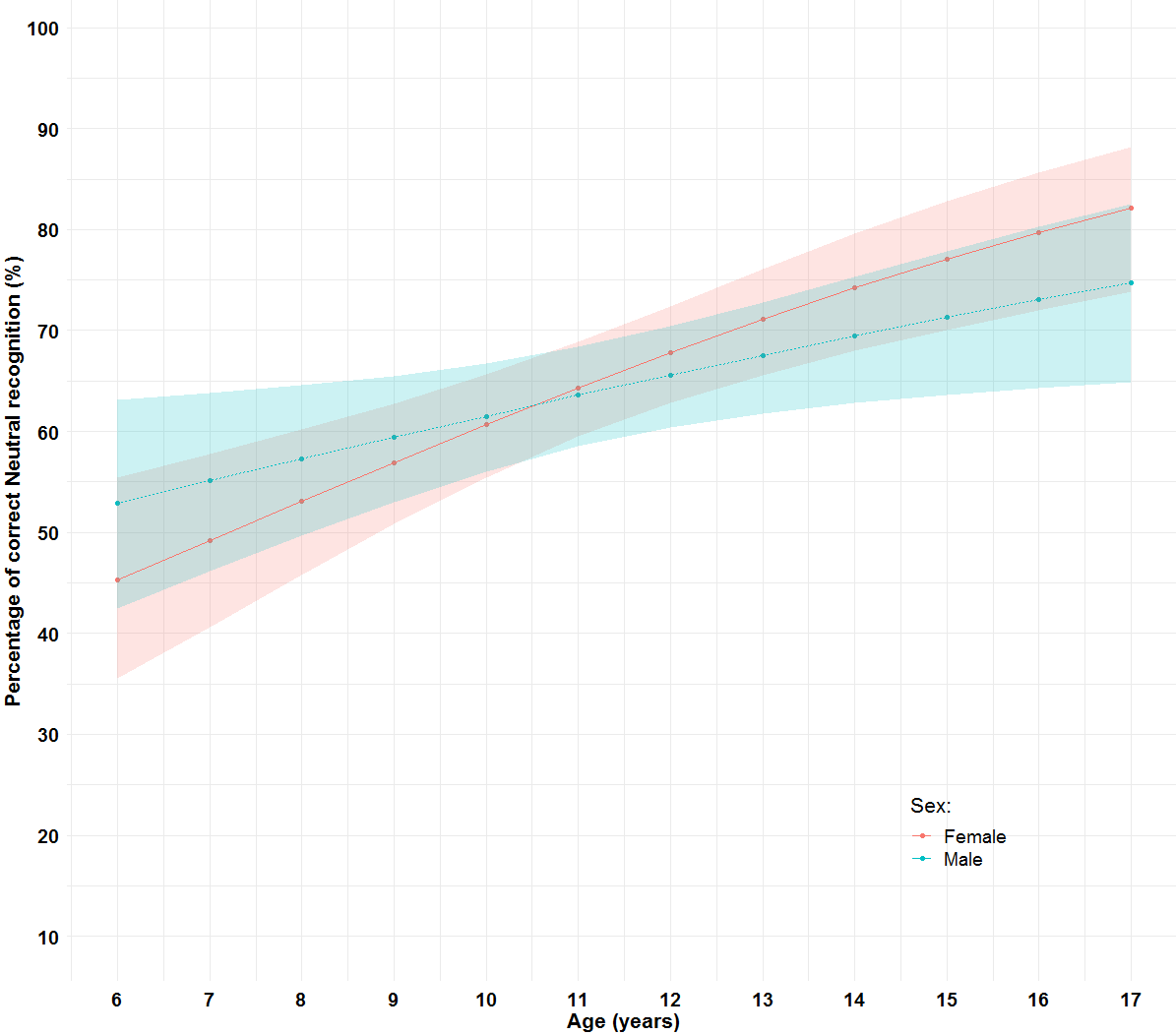


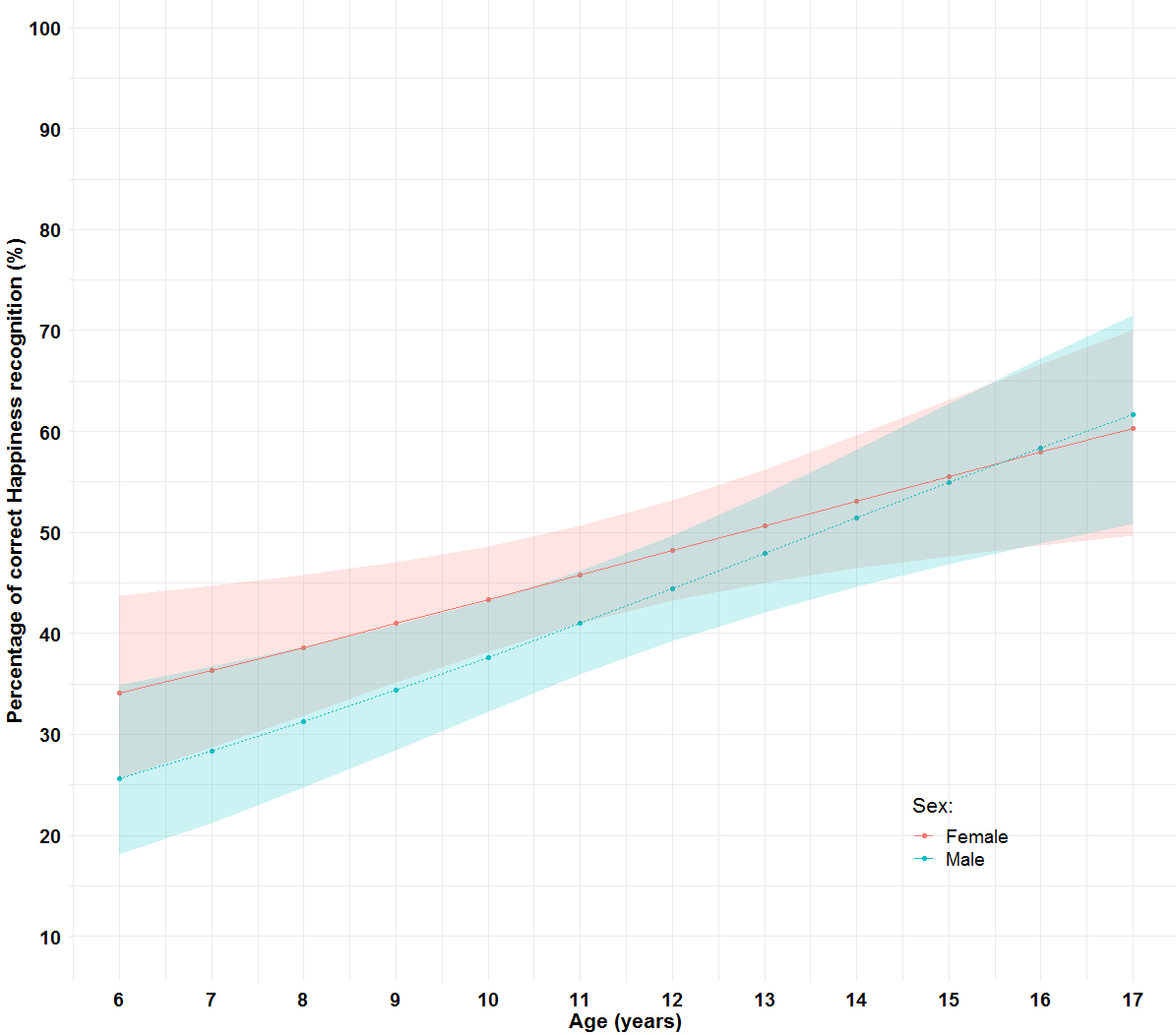

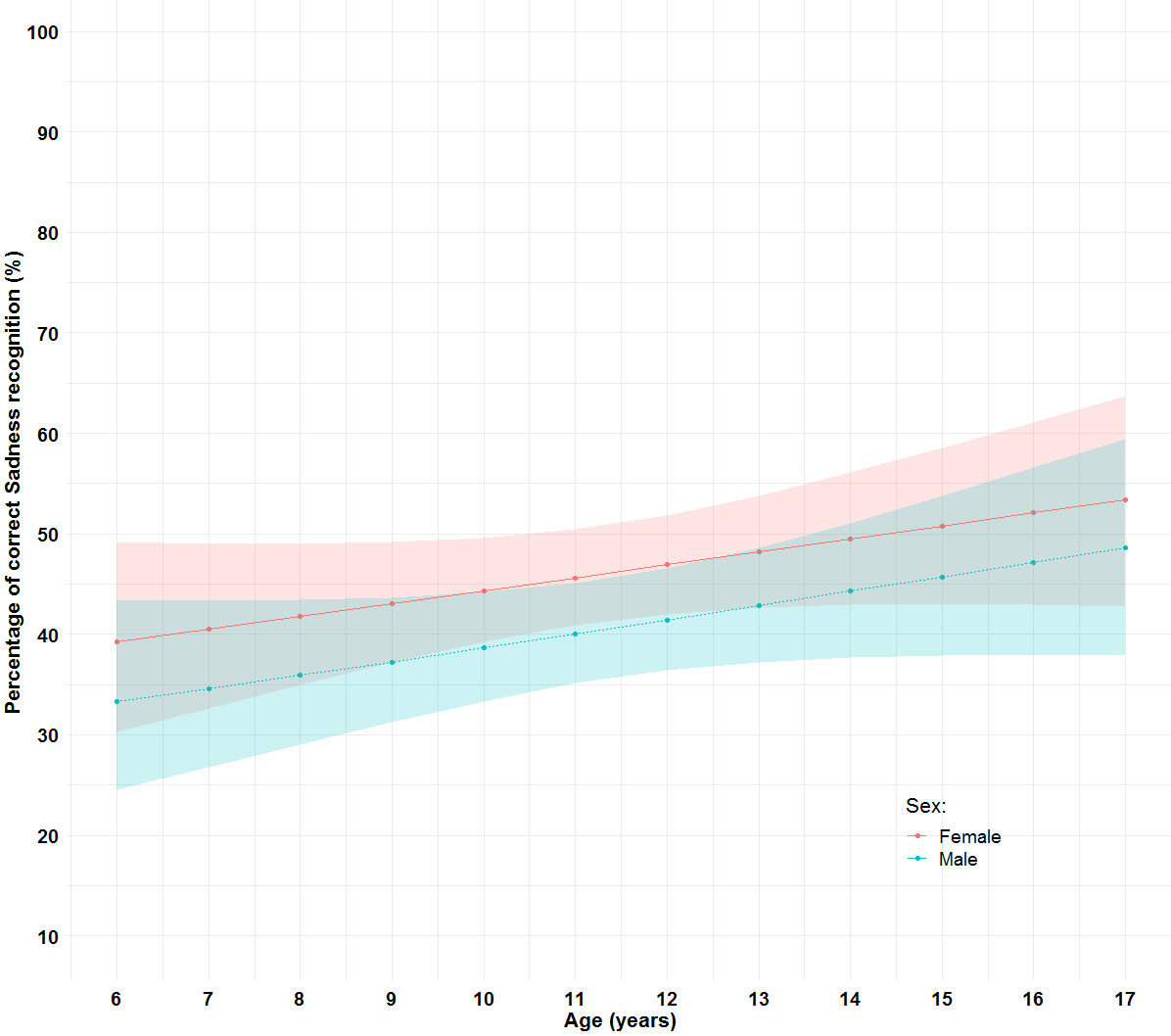


**Supplementary audio material**

Supplementary Audio 1 : **Audio example 1, Joy **

Supplementary Audio 2 : **Audio example 2, Fear **

Supplementary Audio 3 : **Audio example 3, Anger **
